# Supplementary material for: Plant-Based Diets and the Incidence of Asthma Symptoms among Elderly Women, and the Mediating Role of Body Mass Index
Source: Nutrients. 2022 Dec 22;15(1):52. doi: 10.3390/nu15010052 (PMC9824479; doi:10.3390/nu15010052)
Supplement: Supplementary file 1 [file nutrients-15-00052-s001.zip › nutrients-2074628-supplementary.pdf]

nutrients

MDPI

Supplementary Materials

Plant-Based Diets and the Incidence of Asthma Symptoms among Elderly Women, and the Mediating Role of Body Mass Index

Wassila Ait-hadad [1], Annabelle Bédard [1], Rosalie Delvert [1], Laurent Orsi [1], Sébastien Chanoine [2], Orianne Dumas [1], Nasser Laouali [3,*], Nicole Le Moual [1], Bénédicte Leynaert [1], Valérie Siroux [2], Marie-Christine Boutron-Ruault [3] and Raphaëlle Varraso [1]

Supplementary Online Content

- **Method S1**. Baseline characteristics of included and excluded women
- **Figure S1.** Study design and flow chart of participants
- **Table S1.** Baseline characteristics of included and excluded women
- **Table S2.** Scoring criteria for the healthy Plant-based Diet Index (hPDI), unhealthy Plant-based Diet Index (uPDI) and Plant-based Diet Index (PDI)
- **Table S3.** Association between the *healthy* Plant-based Diet Index (hPDI) and the incidence of asthma symptom, mediated by BMI, further adjusted for total energy intake (n = 8621)
- **Table S4.** Association between the *healthy* Plant-based Diet Index (hPDI) and the incidence of asthma symptom, mediated by BMI assessed in 2005 (n = 8621)
- **Table S5.** Association between the *healthy* Plant-based Diet Index (hPDI) and the incidence of asthma symptom, mediated by BMI assessed in 2011 (n = 8621)
- **Table S6.** Association between the *healthy* Plant-based Diet Index (hPDI) and the incidence of asthma symptom, mediated by BMI, after exclusion of women with cancers or CVD at baseline (n = 7140)
- **Table S7.** Association between the *unhealthy* Plant-Based Diet Index (uPDI) and the incidence of asthma symptom, mediated by BMI, further adjusted for total energy intake (n = 8621)
- **Table S8.** Association between the *unhealthy* Plant-Based Diet Index (uPDI) and the incidence of asthma symptom, mediated by BMI assessed in 2005 (n = 8621)
- **Table S9.** Association between the *unhealthy* Plant-Based Diet Index (uPDI) and the incidence of asthma symptom, mediated by BMI assessed in 2011 (n = 8621)
- **Table S10.** Association between the *unhealthy* Plant-Based Diet Index (uPDI) and the incidence of asthma symptom, mediated by BMI, after exclusion of women with cancers or CVD at baseline (n = 7140)
- **Table S11.** Association between the Plant-Based Diet Index (PDI) and the incidence of asthma symptom, mediated by BMI

Method S1. Characteristics of included and excluded women (eTable 1)

Women excluded because of missing asthma data (i.e., no response to the main E3N questionnaire in 2018 or missing data for the asthma symptom score in 2011 or in 2018, n = 9107) were older than included participants (n = 5700). After adjustment for age, excluded participant had slightly higher energy intake, were less physically active, were more often current smokers, had a lower educational level, were less often married, had less often farmer parents, and were more often overweight or obese as compared to included women. When additionally excluding participants because of missing dietary data in 1993 or in 2015 (n = 1676), and after adjustment for age, excluded participants were more often current smokers, had a lower educational level, were less often married, had less often farmer parents and were more often overweight or obese, as compared to included women. Finally, when excluding participants with at least 1 asthma symptom at baseline (i.e., in 2011, n = 2921), and after adjustment for age, excluded participants consumed more energy intake, were slightly more often current smokers, had a higher educational level, were less often married, and were more often overweight or obese.

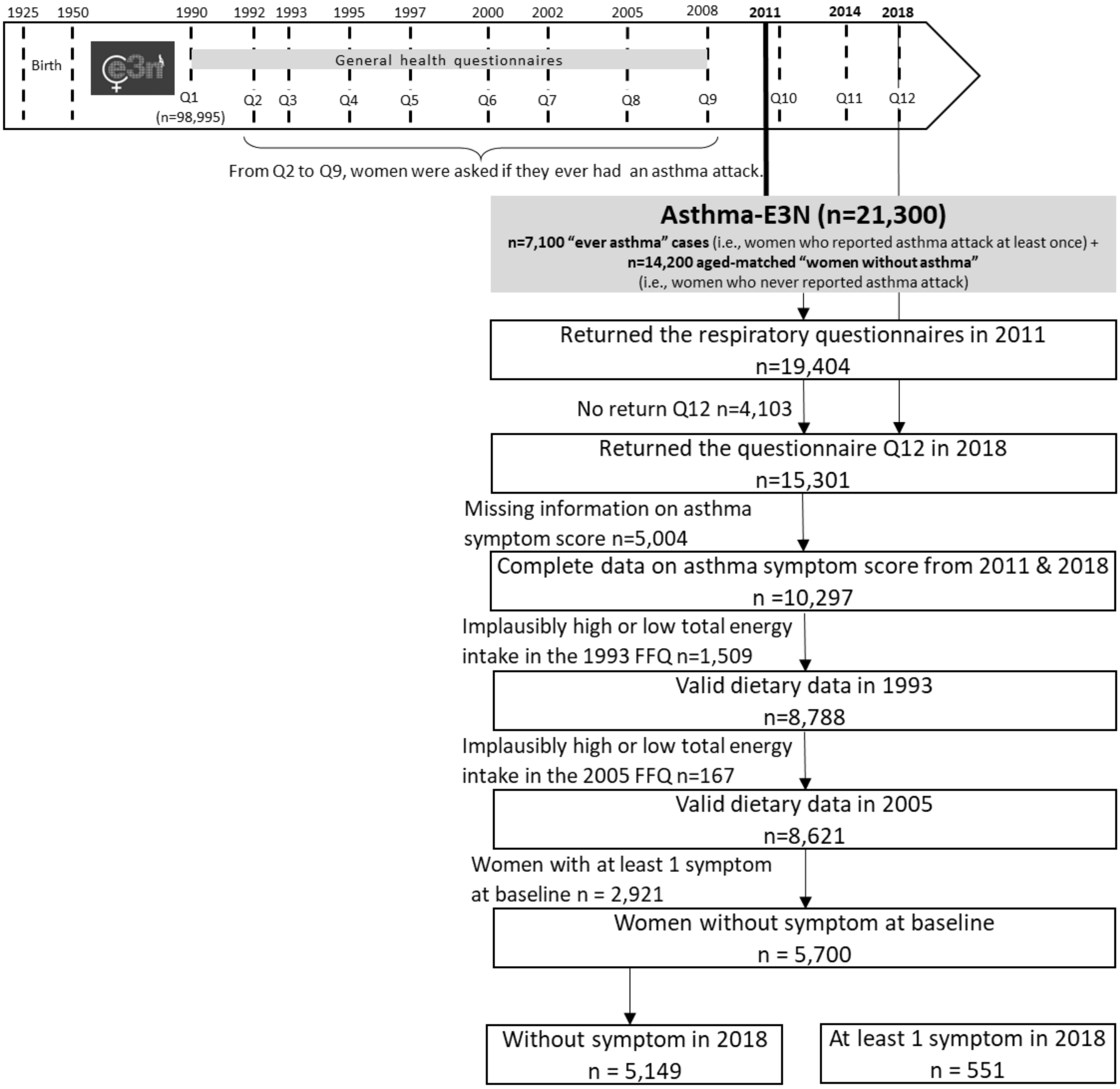

Figure S1. Study design and flow chart of participants.

Table S1. Baseline characteristics of included and excluded women.

	**Included (n = 5700)**	**Excluded asthma data[b] (n = 9107)**	**Age-adjusted p**	**Excluded dietary data[a] (n = 1676)**	**Age-adjusted p**	**Women with symptom at baseline (n = 2921)**	**Age-adjusted p**
hPDI, m (sd)	56.1 (6.0)	52.4 (5.2)	**<0.0001**	/	/	55.4 (6.0)	**<0.0001**
uPDI, m (sd)	52.0 (6.0)					51.4 (6.1)	**<0.0001**
Component score (g/d), m (sd)							
Fiber in whole grains	3.4 (3.3)	3.2 (3.2)	**0.02**	/	/	3.3 (3.3)	0.24
Fruits	288.2 (152.3)	286.2 (160.3)	**0.0007**	/	/	279.6 (155.8)	**0.007**
Vegetables	413.9 (160.0)	415.5 (169.7)	0.08	/	/	419.7 (160.9)	0.18
Nuts	7.3 (9.1)	7.7 (10.0)	**<0.0001**	/	/	7.9 (9.6)	**0.002**
Legumes	24.3 (23.3)	25.1 (26.9)	**<0.0001**	/	/	24.8 (24.3)	0.28
Vegetable oils	24.9 (11.1)	24.2 (11.5)	0.08	/	/	25.1 (11.1)	0.56
Tea and coffee	521.3 (288.6)	519.0 (298.0)	**0.01**	/	/	531.3 (286.2)	0.08
Fruit juices	68.7 (85.9)	72.0 (90.7)	**0.02**	/	/	73.0 (84.5)	**0.03**
Refined grains	173.8 (93.3)	179.9 (100.7)	0.09	/	/	174.1 (90.6)	0.76
Potatoes	65.6 (43.2)	67.9 (48.0)	**0.01**	/	/	67.6 (43.9)	**0.04**
Sugar sweetened beverages	5.4 (21.4)	6.2 (25.4)	**0.004**	/	/	6.4 (22.2)	**0.04**
Sweets and desserts	62.7 (39.8)	62.0 (44.4)	0.22	/	/	61.8 (39.9)	0.26
Animal fat	15.9 (15.7)	16.3 (16.8)	**0.004**	/	/	17.8 (16.6)	**<0.0001**
Dairy	308.2 (169.9)	312.7 (183.9)	0.07	/	/	314.1 (175.5)	0.13
Egg	22.9 (16.8)	25.3 (20.3)	**<0.0001**	/	/	24.2 (17.7)	**0.001**
Fish or Seafood	39.9 (24.9)	41.7 (27.5)	**<0.0001**	/	/	41.5 (26.0)	**0.007**
Meat	104.9 (44.5)	108.7 (51.3)	**<0.0001**	/	/	112.1 (47.3)	**<0.0001**
Misc. animal-based food	74.4 (47.6)	77.1 (54.8)	**<0.0001**	/	/	79.1 (486)	**<0.0001**
Age (years), m (sd)	62.3 (5.6)	65.3 (6.7)		62.3 (5.5)	/	62.5 (5.5)	
Energy intake(kcal/d), m (sd)	2,264 (509)	2,292 (646)	**<0.0001**	/	/	2,307 (521)	**<0.0001**
Leisure-time physical activity, MET/week	62.6 (50.0)	58.4 (50.7)	**<0.0001**	62.4 (52.5)	0.53	62.4 (50.5)	0.79
Smoking status, n (%)			**<0.0001**		0.06		**<0.0001**
Never smoker	3,068 (53.8)	4,778 (52.5)		867 (51.7)		1,428 (48.9)	
Ex-smoker	2,247 (39.4)	3,641 (40.0)		658 (39.3)		1,289 (44.1)	
Current smoker	385 (6.8)	685 (7.5)		151 (9.0)		204 (7.0)	
Educational level, n (%)			**<0.0001**		**<0.0001**		**0.02**
< high school diploma	511 (8.9)	1,314 (14.4)		240 (14.3)		78 (2.7)	
High school to 2-level university	2,839 (49.8)	4,435 (48.7)		773 (46.1)		206 (7.1)	

3- to 4-level university	1,132 (19.9)	1,463 (16.1)		317 (18.9)		1,478 (50.6)	
≥ 5-level university	1,055 (18.5)	1,536 (16.9)		268 (16.0)		583 (19.9)	
Missing	163 (2.9)	359 (3.9)		78 (4.7)		576 (19.7)	
Marital status, n (%)			**<0.0001**		**<0.0001**		**<0.0001**
No	1,361 (23.9)	2,885 (31.7)		511 (30.5)		818 (28.0)	
Yes	4,338 (76.1)	6,219 (68.3)		1,163 (69.4)		2,103 (72.0)	
Missing	1 (0.02)	3 (0.03)		2 (0.1)		0 (0)	
Having farmer parents, n (%)			**<0.0001**		**<0.0001**		0.08
No	4,908 (86.1)	7,673 (84.3)		1,354 (80.8)		2,533 (86.7)	
Yes	668 (11.7)	894 (9.8)		181 (10.8)		310 (10.6)	
Missing	124 (2.2)	540 (5.9)		141 (8.4)		78 (2.7)	
BMI (kg/m²), m (sd)	23.1 (3.7)	24.2 (4.5)	**<0.0001**	23.9 (4.5)	**<0.0001**	24.5 (4.2)	**<0.0001**
BMI (kg/m²), n (%)			**<0.0001**		**<0.0001**		**<0.0001**
< 20	990 (17.4)	1,326 (14.6)		277 (16.5)		324 (11.1)	
[20-25[kg/m²	3,304 (58.0)	4,533 (49.8)		853 (50.9)		1,437 (49.2)	
[25-30[kg/m²	1,117 (20.7)	2,373 (26.1)		400 (23.9)		886 (30.3)	
≥ 30 kg/m²	229 (4.0)	875 (9.6)		146 (8.7)		274 (9.4)	

[a] Excluded women because they did not complete the food questionnaire in 1993 or in 2005, or had an implausibly high or low total energy intake in 1993 or in 2005. [b] Excluded women because they did not respond to the Q12 or answer to the asthma symptom score questions in 2011 or in 2018.

Table S2. Scoring criteria for the healthy Plant-based Diet Index (hPDI), unhealthy Plant-based Diet Index (uPDI) and Plant-based Diet Index (PDI).

Plant food groups	Food items used from diet questionnaires	Score hPDI	Score uPDI	Score PDI
Healthy				
Fibres from cereals	Fibre from breakfast cereal, fibre from bread, fibre from brown rice	+	-	+
Fruits	Raisins or grapes, prunes, bananas, cantaloupe, watermelon, fresh apples or pears, oranges, grapefruit, strawberries, blueberries, peaches or apricots or plums	+	-	+
Vegetables	Tomatoes, tomato juice, tomato sauce, broccoli, cabbage, cauliflower, brussels sprouts, carrots, mixed vegetables, yellow or winter squash, eggplant or zucchini, yams or sweet potatoes, spinach cooked, spinach raw, kale or mustard or chard greens, iceberg or head lettuce, romaine	+	-	+
Nuts	Nuts, peanut butter	+	-	+
Legumes	String beans, tofu or soybeans, beans or lentils, peas or lima beans	+	-	+
Vegetable oils	Oil-based salad dressing, vegetable oil used for cooking	+	-	+
Tea and coffee	Tea, coffee, decaffeinated coffee	+	-	+
Less healthy				
Fruit juices	Apple cider (nonalcoholic) or juice, orange juice, grapefruit juice, other fruit juice	-	+	+
Refined grains	Refined grain breakfast cereal, white bread, English muffins or bagels or rolls, muffins or biscuits, white rice, pancakes or waffles, crackers, pasta	-	+	+
Potatoes	French fries, baked or mashed potatoes, potato or corn chips	-	+	+
Sugar sweetened beverages	Colas with caffeine and sugar, colas without caffeine but with sugar, other carbonated beverages with sugar, noncarbonated fruit drinks with sugar	-	+	+
Sweets and desserts	Chocolates, candy bars, candy without chocolate, cookies (home-baked and ready-made), brownies, doughnuts, cake (home-baked and ready-made), sweet roll (home-baked and ready-made), pie (home-baked and readymade), jams or jellies or preserves or syrup or honey	-	+	+
**Animal food groups**				
Animal fat	Butter added to food, butter or lard used for cooking	-	-	-
Dairy	Skim low fat milk, whole milk, cream, sour cream, sherbet, ice cream, yogurt, cottage or ricotta cheese, cream cheese, other cheese	-	-	-
Egg	Egg	-	-	-
Fish or seafood	Canned tuna, dark meat fish, other fish, shrimp or lobster or scallops	-	-	-
Meat	Chicken or turkey with skin, chicken or turkey without skin, bacon, hot dogs, processed meats, liver, hamburger, beef or pork or lamb mixed dish, beef or pork or lamb main dish	-	-	-
Miscellaneous animal-based foods	Pizza, chowder or cream soup, mayonnaise or other creamy salad dressing	-	-	-

+ : positive scores. **-** : negative scores.

Table S3. Association between the *healthy* Plant-based Diet Index (hPDI) and the incidence of asthma symptom, mediated by BMI, further adjusted for total energy intake (n = 8621).

hPDI	No.	Total effect OR (95%CI)	Direct effect OR (95%CI)	Indirect effect OR (95%CI)	Proportion mediated
Continuous	551/5,149	0.89 (073–1.03)	0.91 (0.76–1.08)	0.97 (0.92–1.02)	18%
Quintile 1	106/1,017	1.00 (ref)	1 .00 (ref)	1 .00 (ref)	
Quintile 2	134/1,138	0.87 (0.65–1.12)	0.88 (0.66–1.15)	**0.98 (0.97–0.99)**	
Quintile 3	98/973	0.91 (0.69–1.19)	0.93 (0.69–1.22)	**0.97 (0.94–0.99)**	45%
Quintile 4	109/1,016	0.89 (0.66–1.19)	0.93 (0.69–1.22)	**0.96 (0.93–0.98)**	
Quintile 5	104/1,005	0.89 (0.64–1.18)	0.94 (0.66–1.25)	**0.95 (0.91–0.98)**	

Ref = referent values. Odds ratio (OR) and 95% confidence interval (CI) were estimated from marginal structural models for an increase of 1 quintile in the hPDI or per ten increments of hPDI; 95% CI were obtained from 500 bootstrapped samples. The total effect represents the overall effect of the exposure (diet) on the disease (asthma); the indirect effect represents the effect passing through the mediator (body mass index); and the direct effect represents the effect unexplained by the mediator. Models were adjusted for age, physical activity, smoking, educational level, marital status, having farmer parents and total energy intake.

Table S4. Association between the *healthy* Plant-based Diet Index (hPDI) and the incidence of asthma symptom, mediated by BMI assessed in 2005 (n = 8621).

hPDI	No.	Total effect OR (95%CI)	Direct effect OR (95%CI)	Indirect effect OR (95%CI)	Proportion mediated
Continuous	551/5,149	0.88 (0.74–1.03)	0.92 (0.77–1.06)	0.96 (0.91–1.01)	47%
Quintile 1	107/969	1.00 (ref)	1 .00 (ref)	1 .00 (ref)	
Quintile 2	108/1,008	0.86 (0.64–1.17)	0.87 (0.65–1.18)	0.99 (0.98–0.99)	
Quintile 3	127/1,119	0.89 (0.67–1.17)	0.88 (0.66–1.16)	0.98 (0.96–0.99)	24%
Quintile 4	110/1,055	0.85 (0.64–1.12)	0.88 (0.66–1.16)	0.97 (0.95–0.98)	
Quintile 5	99/998	0.83 (0.61–1.08)	0.87 (0.64–1.14)	0.96 (0.93–0.98)	

Ref = referent values. Odds ratio (OR) and 95% confidence interval (CI) were estimated from marginal structural models for an increase of 1 quintile in the hPDI or per ten increments of hPDI; 95% CI were obtained from 500 bootstrapped samples. The total effect represents the overall effect of the exposure (diet) on the disease (asthma); the indirect effect represents the effect passing through the mediator (body mass index); and the direct effect represents the effect unexplained by the mediator. Models were adjusted for age, physical activity, smoking, educational level, marital status and having farmer parents.

Table S5. Association between the *healthy* Plant-based Diet Index (hPDI) and the incidence of asthma symptom, mediated by BMI assessed in 2011 (n = 8621).

hPDI	No.	Total effect	Direct effect	Indirect effect	Proportion mediated
		OR (95%CI)	OR (95%CI)	OR (95%CI)	
Continuous	551/5,149	0.86 (0.74–1.01)	0.89 (0.77–1.01)	0.97 (0.93–1.01)	22%
Quintile 1	107/969	1.00 (ref)	1 .00 (ref)	1 .00 (ref)	
Quintile 2	108/1,008	0.85 (0.63–1.11)	0.87 (0.65–1.14)	0.98 (0.95–0.99)	
Quintile 3	127/1,119	0.87 (0.66–1.14)	0.91 (0.69–1.20)	0.96 (0.91–0.98)	26%
Quintile 4	110/1,055	0.84 (0.62–1.09)	0.89 (0.66–1.16)	0.94 (0.89–0.98)	
Quintile 5	99/998	0.85 (0.62–1.11)	0.89 (0.66–1.22)	0.93 (0.87–0.97)	

Ref = referent values. Odds ratio (OR) and 95% confidence interval (CI) were estimated from marginal structural models for an increase of 1 quintile in the hPDI or per ten increments of hPDI; 95% CI were obtained from 500 bootstrapped samples. The total effect represents the overall effect of the exposure (diet) on the disease (asthma); the indirect effect represents the effect passing through the mediator (body mass index); and the direct effect represents the effect unexplained by the mediator. Models were adjusted for age, physical activity, smoking, educational level, marital status and having farmer parents.

Table S6. Association between the *healthy* Plant-based Diet Index (hPDI) and the incidence of asthma symptom, mediated by BMI, after exclusion of women with cancers or CVD at baseline (n = 7140).

hPDI	No.	Total effect	Direct effect	Indirect effect	Proportion mediated
		OR (95%CI)	OR (95%CI)	OR (95%CI)	
Continuous	428/4,440	0.89 (0.74–1.05)	0.91 (0.77–1.07)	0.98 (0.92–1.03)	20%
Quintile 1	89/839	1.00 (ref)	1 .00 (ref)	1 .00 (ref)	
Quintile 2	84/878	0.91 (0.65–1.22)	0.92 (0.67–1.23)	0.99 (0.98–0.99)	31%
Quintile 3	87/963	0.82 (0.58–1.11)	0.84 (0.60–1.13)	0.98 (0.96–0.99)	
Quintile 4	88/915	0.87 (0.63–1.18)	0.89 (0.65–1.27)	0.97 (0.95–0.99)	
Quintile 5	80/845	0.87 (0.62–1.22)	0.91 (0.66–1.27)	0.95 (0.93–0.98)	

Ref = referent values. Odds ratio (OR) and 95% confidence interval (CI) were estimated from marginal structural models for an increase of 1 quintile in the hPDI or per ten increments of hPDI; 95% CI were obtained from 500 bootstrapped samples. The total effect represents the overall effect of the exposure (diet) on the disease (asthma); the indirect effect represents the effect passing through the mediator (body mass index); and the direct effect represents the effect unexplained by the mediator. Models were adjusted for age, physical activity, smoking, educational level, marital status and having farmer parents.

Table S7. Association between the *unhealthy* Plant-Based Diet Index (uPDI) and the incidence of asthma symptom, mediated by BMI, further adjusted for total energy intake (n = 8621).

uPDI	No.	Total effect OR (95%CI)	Direct effect OR (95%CI)	Indirect effect OR (95%CI)	Proportion mediated
Continuous	551/5,149	0.96 (0.82–1.13)	1.03 (0.88–1.24)	0.94 (0.79–1.00)	/
Quintile 1	106/1,017	1.00 (ref)	1 .00 (ref)	1 .00 (ref)	
Quintile 2	134/1,138	1.07 (0.80–1.39)	1.09 (0.82–1.43)	0.98 (0.97–0.99)	
Quintile 3	98/973	0.93 (0.68–1.21)	0.97 (0.71–1.25)	0.96 (0.94–0.98)	83%
Quintile 4	109/1,016	0.95 (0.71–1.38)	1.02 (0.74–1.34)	0.94 (0.91–0.97)	
Quintile 5	104/1,005	0.88 (0.66–1.51)	0.95 (0.72–1.26)	0.93 (0.89–0.97)	

Ref = referent values. Odds ratio (OR) and 95% confidence interval (CI) were estimated from marginal structural models for an increase of 1 quintile in the uPDI or per ten increments of uPDI; 95% CI were obtained from 500 bootstrapped samples. The total effect represents the overall effect of the exposure (diet) on the disease (asthma); the indirect effect represents the effect passing through the mediator (body mass index); and the direct effect represents the effect unexplained by the mediator. Models were adjusted for age, physical activity, smoking, educational level, marital status, having farmer parents and total energy intake.

Table S8. Association between the *unhealthy* Plant-Based Diet Index (uPDI) and the incidence of asthma symptom, mediated by BMI assessed in 2005 (n = 8621).

uPDI	No.	Total effect OR (95%CI)	Direct effect OR (95%CI)	Indirect effect OR (95%CI)	Proportion mediated
Continuous	551/5,149	0.97 (0.80–1.16)	1.04 (0.88–1.23)	0.93 (0.76–1.01)	/
Quintile 1	106/1,017	1.00 (ref)	1 .00 (ref)	1 .00 (ref)	
Quintile 2	134/1,138	1.10 (0.81–1.47)	1.12 (0.82–1.49)	0.99 (0.97–1.00)	
Quintile 3	98/973	0.97 (0.68–1.35)	0.99 (0.68–1.63)	0.97 (0.95–0.99)	/
Quintile 4	109/1,016	0.96 (0.68–1.31)	1.00 (0.71–1.36)	0.96 (0.93–0.99)	
Quintile 5	104/1,005	0.97 (0.69–1.31)	1.02 (0.72–1.39)	0.95 (0.91–0.98)	

Ref = referent values. Odds ratio (OR) and 95% confidence interval (CI) were estimated from marginal structural models for an increase of 1 quintile in the uPDI or per ten increments of uPDI; 95% CI were obtained from 500 bootstrapped samples. The total effect represents the overall effect of the exposure (diet) on the disease (asthma); the indirect effect represents the effect passing through the mediator (body mass index); and the direct effect represents the effect unexplained by the mediator. Models were adjusted for age, physical activity, smoking, educational level, marital status and having farmer parents.

Table S9. Association between the *unhealthy* Plant-Based Diet Index (uPDI) and the incidence of asthma symptom, mediated by BMI assessed in 2011 (n = 8621).

uPDI	No.	Total effect	Direct effect	Indirect effect	Proportion mediated
		OR (95%CI)	OR (95%CI)	OR (95%CI)	
Continuous	551/5,149	0.94 (0.81–1.10)	0.98 (0.84–1.13)	0.96 (0.91–1.01)	67%
Quintile 1	106/1,017	1.00 (ref)	1 .00 (ref)	1 .00 (ref)	
Quintile 2	134/1,138	1.00 (ref)	1 .00 (ref)	1 .00 (ref)	
Quintile 3	98/973	1.10 (0.83–1.48)	1.12 (0.85–1.52)	0.97 (0.97–0.99)	/
Quintile 4	109/1,016	0.94 (0.69–1.26)	0.98 (0.73–1.30)	0.96 (0.94–0.98)	
Quintile 5	104/1,005	0.97 (0.70–1.30)	1.02 (0.75–1.37)	0.95 (0.91–0.97)	

Ref = referent values. Odds ratio (OR) and 95% confidence interval (CI) were estimated from marginal structural models for an increase of 1 quintile in the uPDI or per ten increments of uPDI; 95% CI were obtained from 500 bootstrapped samples. The total effect represents the overall effect of the exposure (diet) on the disease (asthma); the indirect effect represents the effect passing through the mediator (body mass index); and the direct effect represents the effect unexplained by the mediator. Models were adjusted for age, physical activity, smoking, educational level, marital status and having farmer parents.

Table S10. Association between the *unhealthy* Plant-Based Diet Index (uPDI) and the incidence of asthma symptom, mediated by BMI, after exclusion of women with cancers or CVD at baseline (n=7140).

uPDI	No.	Total effect	Direct effect	Indirect effect	Proportion mediated
		OR (95%CI)	OR (95%CI)	OR (95%CI)	
Continuous	428/4,440	0.96 (0.79–1.12)	0.99 (0.82–1.15)	0.97 (0.92–1.03)	75%
Quintile 1	84/868	1.00 (ref)	1 .00 (ref)	1 .00 (ref)	
Quintile 2	104/999	1.07 (0.82–1.39)	1.11 (0.86–1.46)	0.96 (0.90–0.99)	
Quintile 3	78/849	0.91 (0.65–1.23)	0.97 (0.72–1.31)	0.93 (0.85–0.98)	83%
Quintile 4	82/887	0.99 (0.73–1.30)	1.09 (0.77–1.45)	0.91 (0.83–0.97)	
Quintile 5	80/837	0.88 (0.65–1.19)	0.98 (0.73–1.32)	0.90 (0.81–0.96)	

Ref = referent values. Odds ratio (OR) and 95% confidence interval (CI) were estimated from marginal structural models for an increase of 1 quintile in the uPDI or per ten increments of uPDI; 95% CI were obtained from 500 bootstrapped samples. The total effect represents the overall effect of the exposure (diet) on the disease (asthma); the indirect effect represents the effect passing through the mediator (body mass index); and the direct effect represents the effect unexplained by the mediator. Models were adjusted for age, physical activity, smoking, educational level, marital status and having farmer parents.

Table S11. Association between the Plant-Based Diet Index (PDI) and the incidence of asthma symptom, mediated by BMI.

PDI	No.	Total effect OR (95%CI)	Direct effect OR (95%CI)	Indirect effect OR (95%CI)	Proportion mediated
Continuous	551/5,149	0.96 (0.79–1.16)	1.02 (0.85–1.24)	0.94 (0.83–1.00)	/
Quintile 1	109/911	1.00 (ref)	1 .00 (ref)	1 .00 (ref)	
Quintile 2	97/980	0.80 (0.57–1.05)	0.83 (0.63–1.10)	0.96 (0.90–0.99)	
Quintile 3	126/1,161	0.80 (0.48–1.03)	0.86 (0.63–1.10)	0.93 (0.85–0.98)	71%
Quintile 4	110/1,035	0.90 (0.64–1.19)	0.98 (0.72–1.28)	0.91 (0.82–0.97)	
Quintile 5	109/1,062	0.86 (0.64–1.11)	0.96 (0.68–1.34)	0.90 (0.80–0.97)	

Ref = referent values. Odds ratio (OR) and 95% confidence interval (CI) were estimated from marginal structural models for an increase of 1 quintile in the PDI or per ten increments of PDI; 95% CI were obtained from 500 bootstrapped samples. The total effect represents the overall effect of the exposure (diet) on the disease (asthma); the indirect effect represents the effect passing through the mediator (body mass index); and the direct effect represents the effect unexplained by the mediator. Models were adjusted for age, physical activity, smoking, educational level, marital status and having farmer parents.
